# Cleanroom strategies for micro- and nano-fabricating flexible implantable neural electronics

**DOI:** 10.1098/rsta.2021.0009

**Published:** 2022-07-25

**Authors:** Finlay Walton, Maria Cerezo-Sanchez, Eve McGlynn, Rupam Das, Hadi Heidari

**Affiliations:** Microelectronics Lab, James Watt School of Engineering, University of Glasgow, Glasgow, G12 8QQ, UK

**Keywords:** nanofabrication, brain, flexible, electronics, implantable device

## Abstract

Implantable electronic neural interfaces typically take the form of probes and are made with rigid materials such as silicon and metals. These have advantages such as compatibility with integrated microchips, simple implantation and high-density nanofabrication but tend to be lacking in terms of biointegration, biocompatibility and durability due to their mechanical rigidity. This leads to damage to the device or, more importantly, the brain tissue surrounding the implant. Flexible polymer-based probes offer superior biocompatibility in terms of surface biochemistry and better matched mechanical properties. Research which aims to bring the fabrication of electronics on flexible polymer substrates to the nano-regime is remarkably sparse, despite the push for flexible consumer electronics in the last decade or so. Cleanroom-based nanofabrication techniques such as photolithography have been used as pattern transfer methods by the semiconductor industry to produce single nanometre scale devices and are now also used for making flexible circuit boards. There is still much scope for miniaturizing flexible electronics further using photolithography, bringing the possibility of nanoscale, non-invasive, high-density flexible neural interfacing. This work explores the fabrication challenges of using photolithography and complementary techniques in a cleanroom for producing flexible electronic neural probes with nanometre-scale features.

This article is part of the theme issue 'Advanced neurotechnologies: translating innovation for health and well-being'.

## 1. Introduction

Neurological disorders make up the leading cause of disability-adjusted life-years lost (DALYs) and are the second leading cause of death worldwide (9 million in 2016), second only to heart disease [1]. Two of the four most significant contributors to the DALYS are migraines and dementias – the latter of which includes Alzheimer’s disease. These disorders—as well as others such as epilepsy and Parkinson's disease—could benefit enormously from a better understanding of how they manifest themselves in the brain and wider nervous system, in addition to novel treatments and procedures. Electronic neural probes that can record neural signals with high temporal and spatial resolution and stimulate with neuron-level precision have the potential to play a significant role in addressing this challenge now and in the future. This potential is reflected in significant academic research [2–5] and the emergence of countless start-up companies in this field, such as Neuralink [6], Salvia Bioelectronics B. V. and Nyxoah S. A.

**Figure 1.**
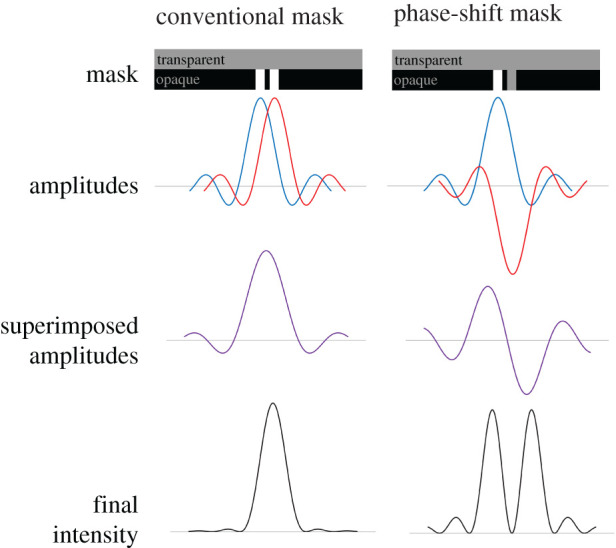
Comparison between how a conventional photolithographic mask and phase-shift mask work with regard to features that are of similar size to the wavelength of light used. Two neighbouring light spots in a conventional mask lead to a single large spot, as diffraction means that the two spots cannot be discriminated. In the phase-shift mask, a transparent element is added to shift the phase of one of the spots, leading to destructive interference in the space between the spots, resulting in two spots which can be smaller than the limit described by the Rayleigh criterion. (Online version in colour.)

**Figure 2.**
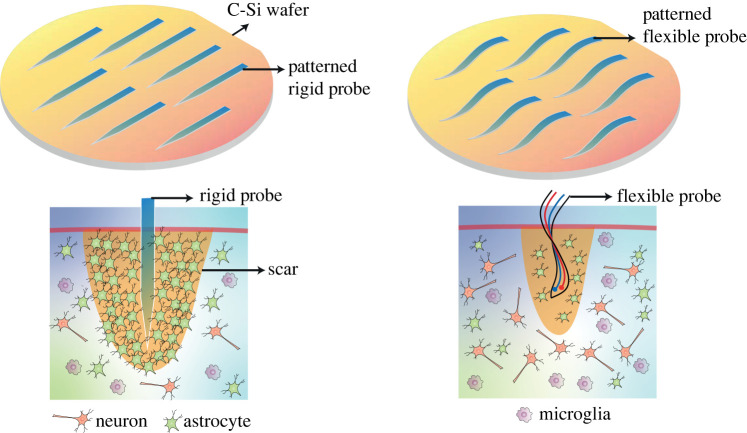
Illustration of the difference in immune responses when traditional rigid probes are used for neural recording/stimulation versus flexible probes. The rigid probes do not conform well with inevitable changes in structure of brain tissue which leads to inflammation and an immune response. (Online version in colour.)

Rigid brain implants like Utah and Michigan [7] style arrays, and the long electrodes used in deep brain stimulation [8], have advantages in terms of fabrication quality, resolution and consistency, but do not conform to the brain shape and are typically made of less biocompatible materials, which can lead to immune responses and inflammation (illustrated in figure 2), in the form of the activation of immune cells such as astrocytes and microglia, leading to glial scarring, device failure and neuronal cell death.[9] This is particularly the case if devices are used for long-term implantation which is common in medicine due to the prevalence of chronic conditions like Parkinson's disease and severe tremor. Devices that do not trigger this response could be implanted for longer, meaning less frequent invasive surgeries and a lower likelihood of surgery-related complications such as staph infections [10]. Devices on flexible substrates have been shown to produce much lower immune responses (illustrated in figure 1), including less inflammation, normal cell morphology and less damage to the blood-brain barrier (BBB) during insertion [11]. A comparison between rigid and flexible substrates is presented in table 1.

**Table 1.** Comparison between the use of traditional rigid materials like crystalline silicon versus flexible polymer-based materials as substrates for electronic neural probes.

	C-Si	flexible substrate
surface roughness	nanometre range [12]	roughness in nm range [13]
electronic properties	dielectric constant 11.7	variable dielectric constant e.g. 2.44–2.81 [14]
foreign body response	astrocyte activation, glial scarring [15]	reduced or negligible FBR with less neuronal death [16]
blood-brain barrier damage	significant acute inflammation [17]	reduced after 8 weeks of insertion [11]
Young's Modulus	169 GPa [18]	3.2 GPa [3,19]
expected buckling force	2.9 mN [19]	0.25–1.25 mN [20]
typical probe thickness	20 µm [21]	15 µm [22]

Modern electronics have only been scalable down to single nanometre scales because of the high purity, defect-free, flat and rigid substrate surfaces. Fabricating miniaturized electronics on flexible substrates is understandably challenging, as the electronic response changes are bound to change with mechanical stresses induced by flexibility. It is nevertheless possible to apply advanced fabrication techniques such as photolithography to flexible devices. For example, Liu *et al.* produced a device made of a spin-coated polyimide and SU-8 substrate with 25 µm Cr-Au wiring using photolithography [23]. Elon Musk's neuroscience start-up Neuralink use photolithography to produce gold on polyamide ‘threads’ which have diameters between 5 and 50 μm with submicron resolution [6]. Despite the advanced nature of research into polymers and nanofabrication, the niche of nanofabricating on flexible substrates is, in our opinion, underdeveloped. In this work, the focus will be solely on photolithography rather than other pattern transfer methods such as electron-beam lithography and nanoimprint lithography for three reasons: (1) the simplicity of the technique, (2) its wide adoption and (3) the remarkable fact that the Rayleigh criterion (equation (1.1)) has been overcome to produce single nanometre features, leading to Moore's law continuing to apply for half a century [24]. The criterion is the general limit for the minimum resolvable detail in imaging or the minimum distance *D* at which two light spots of wavelength *λ* can be resolved

1.1 $$D=\frac{1.22\lambda }{2n\sin\theta }=\frac{0.61\lambda }{2\text{NA}},$$

where *n* is the refractive index of the medium, *θ* is the extreme angle from the objective lens to the sample. *nsinθ* is often exchanged for numerical aperture (NA), a useful parameter for describing lenses. It is overcome by using *phase-shift masks*, which unlike conventional masks that simply block or transmit light in a given pattern, take advantage of the destructive interference between neighbouring light spots with a phase delay of approximately π. Figure 1 shows how a phase-shift mask can produce light patterns on the substrate with smaller features than could be produced with a conventional mask.

As such, this paper will detail strategies for overcoming some of the critical issues holding back this field, which has enormous potential for helping produce long-term low-invasiveness. These biocompatible probes could improve the quality of life for people who suffer from neurological disorders.

## 2. Material choice

Before fabrication can begin, a substrate material must be chosen, keeping several parameters in mind. Firstly, substrate materials that have an existing body of research behind them in micro and nanofabrication and/or are commercially deployed are an excellent place to begin, including materials such as Parylene C, polyimides, SU-8 and PDMS. Certain polyimides and SU-8 are photo-definable, which can simplify the process steps or reduce them in number—we will come back to this later. For implantable neural probes, the mechanical properties such as Young's modulus of the material must be as close to that of brain tissue as possible (the order of kPa), but not so flexible (less than the order of GPa) that they cannot pierce the tissue or buckle during an attempt at insertion [25]. This conundrum is challenging to solve with material choice alone (Young's modulus of typical flexible substrates is in the single GPa range [3]), so water-soluble stiffeners such as dextran [26] and sucrose [19] are often used during insertion so that the most flexible materials may be implanted successfully without probe damage. The stiffener will then dissolve, often with a timescale that can be controlled with coating thickness or the molecular weight of polymers like dextran [26]. Furthermore, it is essential to make sure the chosen material is magnetic resonance imaging (MRI) compatible. This can be done by avoiding any ferromagnetic materials and using materials (e.g. gold) that have a similar magnetic susceptibility to that of the brain [27].

Most substrates that are used in the semiconductor industry like high-quality crystalline silicon or sapphire require energy-intensive and time-consuming production methods like the Czoralski, edge-freeze or Kyropoulos techniques, followed by lengthy and precise cutting and polishing. Flexible substrates on the other hand are solution processible, which means they can be dissolved in a solvent mixture, spin-coated for a precise film thickness, then baked and cured in a matter of minutes or hours. Spin coating is routinely used in the semiconductor industry to rapidly lay down high quality homogenous layers of solvent processable materials like resists that are commonly 0.1–10 µm thick after baking [28]. To produce approximately 100 µm thick layers for stable tissue implantation, more viscous resists can be used, otherwise many cycles of spinning and baking would be necessary to produce the desired thickness. It is worthwhile considering the baking and curing temperatures of a flexible substrate. SU-8 formulations tend to ‘soft bake’ at less than 100°C and ‘hard bake’ at less than 250°C in under one hour [29,30], but certain polyimides for example require longer bakes with higher temperatures in order to drive out solvent. For example, the widely used HD4100 series of photodefinable (sensitive to UV light, so can be used for photolithography) polyimides require a ramped 375°C cure for up to 3 h to drive out all remaining N-methyl-2-pyrrolidone solvent, as well as a recommended 700°C furnace cleaning cycle [31], bringing optimal fabrication out of reach of most conventional ovens, increasing fabrication time and decreasing throughput. It should also be noted that around 50% of the layer thickness can be lost during baking due to solvent evaporation, which in turn causes stress build-up, which can lead to ruinous wrinkling in the middle of a fabrication process or bending of the final product, particularly for high-aspect ratio structures like neural probes. Four tried and tested strategies to mitigate this issue is including a stress reduction layer [32], slow ramped baking/curing [33], cycled heating and cooling during baking [30] and multiple baking steps, e.g. pre- and post-exposure [34].

It is obviously desirable for the bulk substrate to be biocompatible, but biocompatibility issues can also be addressed by the device being (mostly or entirely) coated with a thin encapsulating layer which can be chosen from a wider range of materials such an dexamethasone [35] or alginate hydrogels [36]. Removing the biocompatibility requirement of the substrate would also enable a wider choice of materials, and a focus to be on other parameters like mechanical and thermal properties.

### (a) Additive versus subtractive protocols

**Figure 3.**
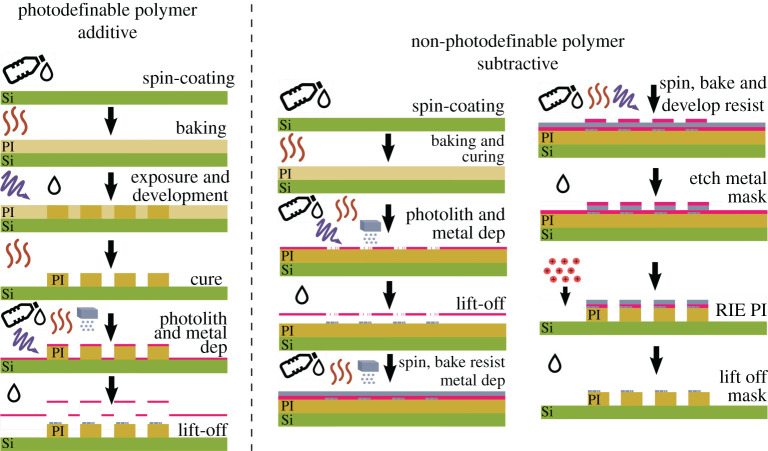
Schematic detailing an example of the process steps required for photolithographically producing electronic circuits on a flexible substrate. (*a*) Using a photodefinable polymer for the probe material typically involves spin-coating it to produce an even film, followed by baking to remove excess solvent, followed by exposure to harden (or break down if it is a positive rather than negative type) the pattern. This may need to be repeated multiple times depending on the desired substrate thickness. It is then normally cured at high temperature to remove the remaining solvent and if needed, complete chemical reactions, e.g. the imidization of polyimides. A second photolithographic step can then be carried out to produce the pattern for the metal pads/tracks/electrodes, followed by metal deposition via sputtering/vapour deposition and lift-off. (*b*) Using non-photodefinable polymers involves carrying out similar processes, but the undesirable polymer is removed via an etching process like ‘dry’ reaction ion etching (pictured), or ‘wet’ etching using strong base solutions. The harsh etching process requires a mask to be in place to protect the rest of the sample, which will normally be made of metal like aluminium. This then means an extra photolithographic step to produce the mask, and another step to remove it. PI = Polyimide and Si = Silicon. (Online version in colour.)

The steps of a nano fabrication protocol may generally be classed as either additive or subtractive, although the overall process is often a combination of the two for producing multi-layered structures requiring complex techniques. As the name suggests, additive methods involve deposition of a new layer such as a polymer, passivation layer or metal. Subtractive protocols remove an existing layer, or part of a layer, typically using chemical (wet) or dry (plasma) etching. A summary of general procedures is depicted in figure 3. Many photoresists including the epoxy-based SU-8 series (Microchem) and HD4100 polyimide series (Dupont) are photo-definable, and as a consequence, they can be processed additively with fewer steps, without the need for harsh processes like plasma etching, which require etch masks. An illustrative example of the difference is depicted in figure 2, which features the typical process steps that are required for producing metal patterns on a patterned photo-definable or non-photo-definable polyimide substrate. The benefits and drawbacks of these contrasting techniques will be discussed herein with regard to producing electronic circuits on miniaturized flexible substrates. A specific example, comparing photo-definable and non-photo-definable polyimides, is provided alongside more general approaches for other polymer substrates such as Parylene C or polydimethylsiloxane (PDMS).

### (b) Polymer deposition

Thin polyimide films may be produced by spin-coating poly (amid acid) precursor onto a clean substrate, generally silicon or glass. Spin-speed curves for commercial precursors are well-documented; however, when the viscosity of the liquid polyimide is known, the theoretical thickness value may be predicted with the following equation

2.1 $$h(t)=\frac{h_{0}}{\sqrt{1+4(\rho \omega^{2}/3\eta )h_{0}^{2}t}},$$

from [37]. The initial thickness at time *t* = 0 is *h*_0_, while the fluid density *ρ* and absolute viscosity *η* are properties of the polymer. Controlled spin speed is denoted by *ω*. The final thickness *h*(*t*) of the film after curing may be determined with a contact profilometer which measures the step height between the substrate and polymer film. Bubbles, contaminants in the precursor, or particles which land on the surface can cause defects in the film, generating comets during the spinning process. Despite this, in a carefully controlled clean room environment, spin-coated films will encounter fewer contaminants and typically have reduced surface roughness compared to pre-prepared commercial films [13]. Edge bead may occur, with a ring of thick polymer around the outside of the wafer, which may be removed with a short high-speed spin at the end of the spin-coating process, or by adapting photomask designs such that only the centre portion of the film is used as a circuit substrate.

Kapton film produced by Dupont is the most famous polyimide, used to create flexible printed circuit boards (PCBs) with a copper layer on one side. Due to its weak adhesion with untreated polyimide [38], copper may be coupled with an intermediary metal layer such as titanium, chromium or aluminium.

## 3. Photoresist

Positive photoresists are incredibly popular as they can produce high-resolution features, with excellent thermal stability [39], and are typically developed in alkaline solutions. By contrast, negative photoresists are prone to distortion during development. Since UV exposure hardens sections of the negative photoresist, leaving unexposed areas soluble, the entire photoresist layer is vulnerable to the developer. The cross-linking which occurs during UV exposure begins from the surface of the photoresist down to the substrate, which means that prolonged exposure is required to harden the resist fully, increasing the possibility of scattered UV radiation and compromising the pattern resolution [40].

**Figure 4.**
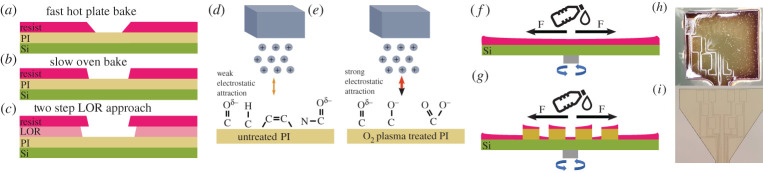
(*a*–*c*) Three processes options in photolithography. Hot plate baking is faster, but often produces more sloping sidewalls than an oven bake will. Metal lift-off is aided by producing a resist bilayer of the resist, plus a ‘lift-off resist’ or LOR. (*d*,*e*) example of surface bond activation for improved metal adhesion to a polyimide surface, (*f*,*g*) uneven resist profiles on thick existing structures, (*h*,*i*) comparison between using additive and subtractive processes for producing metal electronics on polyimide probe shapes respectively - (*h*) has a highly uneven resist profile due as it suffers from the issue shown in (*g*). (Online version in colour.)

In any case, the baking times of the resist should be closely controlled to prevent damage to the layer. Cracking or delamination of the photoresist will be detrimental to the final pattern regardless of whether the photoresist is used as an etch mask, or a lift-off layer [41]. Bilayers that incorporate a lift-off resist (LOR) underneath the top photoresist layer are useful to produce an undercut (figure 4*c*) which encourages clean metal features. The sidewalls of the resist layer can be sloped if the temperature and bake time is not fully optimized, which means the resist stripper will not be able to effectively dissolve the photoresist protected by the metal. An undercut produced by LOR prevents the metal layer from creating a seal over the whole surface of the wafer. Novel etching methods, employing photoresist as an etch mask, use sloped resist sidewalls to control the etch profile of lower layers [42]. Particular care must be given to photoresist spinning on patterned substrates, as depicted in figure 4*f*. The topology of flexible substrates, combining inherent surface roughness with etched/UV-defined probe shapes, may produce uneven resist profiles, further complicating the photolithography process. The order of process steps is equally as important as the success of each individual process.

## 4. Polymer patterning

The additive approach of polymer patterning recruits a photo-definable polyimide which may be simply patterned with a UV exposure step before development removes the unwanted polymer. An in-depth review of the topic is provided in Chapter 5 of [43], detailing the synthesis methods of photosensitive polyimides. A popular option is the HD-4100 series from HD Microsystems, which is negative in tone [44–47]. After UV exposure the polyimide is developed in cyclopentanone and rinsed in propylene glycol methyl ether acetate (PGMEA) at room temperature. Compared to positive tone photoresists, the negative polyimide requires a long exposure time on the order of several minutes, which must be tuned based on the polyimide thickness and feature size. Increased exposure time will ensure the adhesion of small features on the substrate. During development, the soluble polyimide forms a white milky substance before it can be rinsed away, which aids the researcher in determining the reaction end point. Negative tone polyimides may be sensitized through one of the following compositions: ester bonds, ionic bonds, acrylic monomers or photosensitizers such as naphthoquinonediazide, among others. A full discussion of the chemistry is provided in Chapter 5 of [43].

Prior to curing, certain polyimides such as PI-2545 from HD Microsystems may be wet etched in alkaline developer using photoresist as an etch mask. By contrast, after full curing of a polyimide film, it is invulnerable to most solvents, requiring aggressive stripping techniques such as 49% hydrofluoric acid or hot sulphuric acid to remove the polymer from a silicon wafer (PI2545 Product Bulletin from HDMS).

For decades, oxygen plasma has been used to ablate polyimide layers in a reactive ion etch (RIE) process [48,49]. The etch rate may be increased with the inclusion of fluorine-containing gases such as CF_4_ and SF_6_ [50], which boost the concentration of oxygen atoms in the plasma as the etch gases react [51]. Fluorine is added to saturated carbon groups in the polyimide, breaking double carbon–carbon bonds after which point the oxygen atoms break the polymer chain [52], etching the polyimide from the surface. Hard etch masks are required for this dry etch process, as thin photoresist layers lack the etch selectivity required to allow the polyimide layer to be etched before the mask itself is etched away completely. Examples of etch mask materials include aluminium [53], chromium [54], titanium [50], nickel [55] and gold [56], although in some cases thick photoresist may also be used.

### (a) Metal deposition

**Figure 5.**
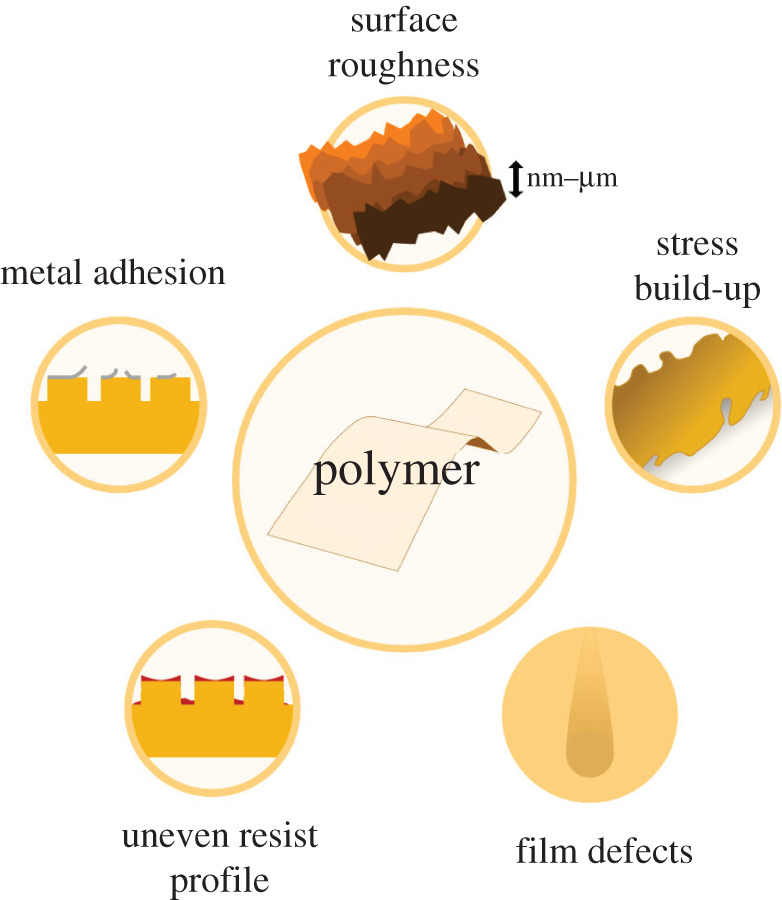
Visual summary of the challenges faced when fabricating electronic probes on flexible polymer substrates in a cleanroom setting using photolithography. (Online version in colour.)

Lift-off is the primary photolithographic method for metal patterning. Photoresist coats the entire sample, and then through UV patterning and development, is removed in the areas intended to be metallized. When a layer of metal is evaporated or sputtered across the whole wafer, it can only make contact with the substrate in the areas the photoresist is not. As described in the previous section, the resist stripping step is not infallible. If the photoresist pattern is imperfect, under- or over-developed, then it is possible the metal will not adhere to the surface or remain in unwanted areas. This is particularly challenging with polymer substrates, as their surface roughness introduces uneven resist profiles. For many metals, the adhesion to polyimide is very poor, and the surface requires plasma treatment to increase its roughness in preparation for metal deposition [57], depicted in figure 4*d*,*e*. For additive style processes that are depicted in figure 3, it may be the case that the probe shape is defined first, making the thickness of the probe an order of magnitude or two greater than the thickness of any subsequent resist coatings. This presents a problem for obtaining even resist profiles, as the path of the resist away from the centre of the sample will likely be impeded by the probe structure. An experimental example of this issue is depicted in figure 4*f*–*i*. Resist thickness varies across the probe shape in figure 4*h*, leading to incorrect exposure doses, followed by simultaneous incomplete and overdevelopment of the resist in different parts of the probe. In this sense, a subtractive process may be advantageous where the probe shape is etched away later, allowing the higher resolution metal patterns to be put down on a flat surface figure 5.

Alternatively, photoresist may be used as an etch mask for a metal layer that covers the entire surface of the wafer in a subtractive process. Much in the way a hard mask protects the underlay during dry etching, the photoresist prevents a liquid etchant from attacking the metal on the polyimide surface, while the uncovered metal is etched away.

The substrate/polyimide interface determines the suitability of wet etching in this instance. When polyimide is spun on glass, it may be easily removed by cutting and peeling [58] or peeling after more than 2 h soaking in de-ionized water. However, when spun on silicon, a release or sacrificial layer such as aluminium [45] is generally employed such that after the final fabrication step, the electronics may be removed from their carrier wafer. Wet etchants, which are acidic solutions, can delaminate polyimide from the glass within a few minutes. On the other hand, if the signal metal layer and sacrificial layer are the same, or can be etched with the same acids, then the probes will delaminate during the metal patterning etch step.

### (b) Heterogeneous bonding to flexible substrates

The integration of soft neural probes with rigid electronic components, for example, PCBs, is a key step in the overall functionalization of brain implantable devices since within these PCBs we can find essential components, ranging from capacitors to memristors, that allow the neural probe to work according to the function desired.

Wirebonding is a common and cost-effective option but is limited in that the minimum wire size of a typical commercially available laboratory wirebonder is 10–20 µm. Wires are generally bonded protruding at a high angle to the plane of the substrate and then encapsulated, limiting how thin the device can be. Wedge bonds offer a lower profile alternative to perpendicular ‘ball-bonded’ wires.

A study by Stieglitz *et al*. [59], however, presented a variation of the wire bonding technique called the ‘Microflex Interconnection Technique’ that allowed three-dimensional interconnects with biocompatible flexible substrates, something of great interest for neural probe integration research. Nonetheless, the small surface that is dealt with in neural probe research, as well as durability concerns, might still be an impediment when using this technique. One way to increase the durability of wire bonding is to encapsulate the wires with non-conductive epoxy.

Conductive epoxy mixtures which contain metallic elements have also been studied by various groups to create interconnections [23,58,60]. A comprehensive study by Yoo & Meng [61] showed that conductive epoxy-bonded interconnections between rigid and soft polymer samples demonstrated the greatest yield strength of the five studied methods, which included wire and bump bonds. A recent study published by Asad *et al*. [62] describes a novel method of ‘pasting and cutting’ GaN µLEDs from a sapphire substrate onto a flexible one using a laser. They carried out a two-step method consisting of first bonding the sapphire substrate containing the µLEDs onto a carrier substrate through spin-coating an adhesive layer that would be removed later through dry-etching, and then applying a laser to lift the µLEDs off the sapphire. The µLEDs were then coated with a conductive layer, flipped and bonded onto the flexible substrate, with the carrier substrate being released. The optoelectronic properties of the µLEDs were unaffected by the transfer process. The method has applications in fabricating optogenetic probes but is yet to be put into practice. One innovative technique was introduced recently in the previously mentioned study conducted by Yoo & Meng [61]. This bonding method called polymer ultrasonic on bump or PUB entails a pre-treatment of the rigid chip with a gold bump, then alignment and finally compression through ultrasonic force, which would finalize the bonding of the rigid chip to the soft substrate, in this case Parylene. The authors demonstrated that compared to other bonding methods, such as wire bonding and epoxy, PUB interconnections showed better performance in terms of electrical parameters, with very low resistance being exhibited in the bonds. It should be noted that these bonds did not perform as well as other bonding methods such as wire bonding when subjected to cycled mechanical tests and pull testing [61].

Overall, integrating a rigid chip with a soft substrate is a crucial step in the fabrication of neural probes and should be carefully studied. Depending on the circumstances of the research, one could argue that wire bonding might be the best method to use for preliminary studies because of its simplicity and cost-effectiveness. Nonetheless, for more advanced stages where *in vivo* implantation is being considered and biocompatibility is essential, other methods such as medical-grade epoxy encapsulation might be considered given that they have been widely studied and offer very adequate results both mechanically and electrically. In the coming years, more research will be conducted on more recently presented methods like laser and PUB, which are showing very promising results and should not be disregarded.

## 5. Chip thinning methods

An additional fundamental step in the fabrication of neural probes is the thinning of the electronic components. When dealing with brain tissue, it is incumbent to obtain good integration between the neural probe and said surrounding tissue. This is achieved through various steps previously mentioned, such as the material selection and the bonding of the soft substrate with the rigid electronic components that control the neural probe. Nonetheless, there is an additional challenge in this case, and that is the size and flexibility of said electronic components. Research has been conducted to produce miniaturized components on silicon chips, among other materials, but the rigidity of silicon chips remains an obstacle. This is where chip thinning comes into place.

Chip thinning methods were first described within the aerospace sector back in the 1960s, during the space race, where a study was published regarding the use of thin silicon wafers for solar cells [63]. However, it has not been until the last three decades that there has been a clear increase of interest within the literature for flexible, high-performance electronics, achieved through chip thinning techniques. In an extensive review presented by Gupta *et al*. [64], the most prominent, as well as more novel methods of chip thinning were described. Based on this, the more relevant methods for neural probe fabrication, all based on silicon wafers, were identified and are presented as follows.

The first method and perhaps the most well-established one would be that of back grinding. This technique consists of a coarse-grinding step, followed by a fine-grinding one, done on the non-active back of the chip by a grinding wheel. The chip is stuck in place by an adhesive tape, which as mentioned in [64] has an effect on the total thickness variation. This technique has the advantage of being cost-effective, very rapid and has been shown to achieve very fine thicknesses of down to 3 µm [65], although it can cause some damage to the chip in the form of warping and breakage during manipulation of the chip. Some could also argue that given that neural probes are very delicate structures, such a technique should not be considered. However, if one were to perform chip thinning before the integration step of the soft substrate with the more rigid electronics, the risk of damage to the neural probe could be attenuated. In fact, a study performed by Sacher *et al*. [66] demonstrated that back grinding could be a suitable chip thinning method for photonic neural probes with a thickness ranging from 50–92 µm. In addition to this, there have been techniques explored to reduce mechanical damage to the chips caused by back grinding, such as the one explored by Morcom *et al*. [67], known as taiko, where grinding is not performed on the periphery of the back, non-active side of the wafer, leaving a ring structure that enhances the structural integrity of the wafer.

The second method has been gaining more interest within the literature in recent years. It is the process of chip thinning through etching, which can be done in two ways: dry etching or wet etching. In terms of dry etching, there are different types, mainly RIE, deep reactive ion etching (DRIE) and physical ion etching (PIE). The first two types use plasma to chemically remove material, in this case, a layer of wafer or substrate, whereas PIE, as its name indicates, physically removes said layer using high energy beams of ions, photons or electrons [68]. There is a tendency for research groups investigating the fabrication of neural probes to use DRIE for thinning processes [21,69], hence for the purpose of this paper, the main focus was on this technique. The main advantage that using this technique provides instead of using others such as back grinding is the fact that it is not as physically aggressive and hence, in terms of the structural integrity of the wafer, the probability of it being compromised is extremely low. In addition, this technique has been proven to work for a variety of thicknesses, even going down to 2 µm in neural probes [69]**,** which makes it incredibly versatile, while still maintaining good mechanical properties. However, it is safe to note that it is a more expensive, complex and long process and that there is a chance of contamination, so it should be taken into account during the selection process, in particular during prototyping and initial experimental stages of chip thinning.

On the other hand, there is wet etching, which has been described in [64] as a technique that requires both pre- and post-processing, unlike the two other techniques already described. The methodology would be as follows: first deposit a mask layer on the back side of the wafer, process the front side and then etch the back side. The advantages of using such a technique would be that, firstly, it is a well-established technique, with studies being done on neural probes as early as 1997 [70], meaning that there is substantial literature to compare to when performing experiments. Moreover, as reported in the literature, low thicknesses of approximately 15 µm have been achieved by Dahiya *et al*. [71] in flexible substrates using tetramethylammonium hydroxide (TMAH) as the etchant, demonstrating its potential for translation to neural probe scenarios, where low thicknesses are desirable. However, one should note that this technique also poses some challenges such as the fact the wafer could be too sensitive to the etchant concentration [72].

Furthermore, in more recent years, more research studies have been published on other methods of chip thinning such as layer transfer methods and epitaxial-based wafers, which will be briefly introduced. The former set includes methods such as proton-induced exfoliation which uses a beam of hydrogen ions to, as its name suggests, exfoliate a layer of the wafer when they are heated. This method, however, has been deemed unsuitable currently because it causes too much damage to the electronic components [64,73]. The latter method of epitaxial-based wafers includes techniques such as the one created by ChipFilm Technology [74] which entails the use of two porous layers, each of a different porosity, between the silicon epitaxial layer and the substrate. By lifting off the epitaxial layer, thinning is achieved. With this method, thicknesses of down to 15 µm have been obtained, as shown in [75]. However, warpage has been reported [76] and there are no studies found by the authors done on neural probes currently, so more research should be conducted on this method.

The process of chip thinning is a crucial one in the fabrication of neural probes, hence the selection of the methodology to follow for such a step must be carefully considered. It is undeniable that the process of back grinding is the simplest, most cost-effective and could prove most useful during initial stages of experimental testing. However, when carefully examining the literature one can see that different processes should be tested according to the application and the geometry of the neural probe. Dry etching seems to be the process that offers the greatest amount of thinning without compromising the integrity of the mechanical or electrical structure as much as the other methods, hence why most research groups perhaps tend to gravitate toward using this technique over the others.

## 6. Future perspective

Truly single nanometre scale devices such as transistors are routinely fabricated on a very large scale in state-of-the-art foundries and are present in the central processing units, memory and other components of devices such as smartphones all over the world. The technique behind this is of course photolithography, which has stretched the limits of the Rayleigh criterion (equation (1.1)). Computational photolithography methods such as phase-shift photomasks and optical proximity correction [77] take advantage of diffraction to go smaller than the criterion would suggest. This staggering miniaturization has been possible in part due to the almost atomic flatness, crystallinity and purity of substrates like silicon, gallium arsenide, sapphire and others. In this aspect, polymer films will never be able to quite reach the same limits due to a lack of crystallinity, the larger size of the polymer fibres and movement of the fibres during flexing. There is still however—to quote the now cliché from Richard Feynman—plenty of room at the bottom [78]. The fact that a technique as simple as spin-coating can be used to produce homogeneous films with values of rms surface roughness of less than 1 nm [53] bodes well for further miniaturization into the nano-regime. The solution processability means that very large-scale integration (VSLI) is possible if applications are widely adopted. Providing that whatever flexing required is consistent, the design, materials and fabrication protocols can be optimized appropriately, allowing for further miniaturization. Although this work has focussed on metals—as their high conductivity lends itself well to micro- and nanoscale electronics—modern conductive polymers which can flex with the substrate may have a role to play instead but must have low enough impedance for devices to be efficient and prevent heating and device or tissue damage [4].

Integration with external components such as power sources, resistors, transistors, diodes and so on is perhaps the greatest challenge due to the mismatch between properties, and their size. There is however progress with miniaturization and increasing efficiency of electronics devices such as µLEDs [79], which will be crucial for converting optogenetics from a tool for in-vivo animal neuroscience studies into a common medical treatment.

## 7. Conclusion

In this work, cleanroom-based strategies for overcoming some of the key issues holding back photolithographic fabrication of neural probes on flexible substrates have been described. This includes additive versus subtractive protocols, metal adhesion, uneven spin-coated films, surface roughness and inhomogeneity. Based on current technology, it is unlikely that flexible electronics will ever be fabricated with the resolution and high-density that is now the standard in the semiconductor industry for rigid substrates, for reasons that we have laid out. There is however still a lot of scope for improvement and miniaturisation using innovative improvements in photolithography and associated cleanroom processes.

## Data Availability

This article has no supporting data.
